# HLA-mismatched stem cell microtransplant prolonged overall survival and promoted immunological reconstitution for multiple myeloma

**DOI:** 10.3389/fimmu.2025.1509588

**Published:** 2025-04-04

**Authors:** Yangyang Lei, Bo Cai, Zhiqing Liu, Anli Xie, Jianhui Qiao, Yi Wang, Xinrui Chen, Fei Peng, Yingxin Zhao, Jiaxin Chen, Wei Guan, Changlin Yu, Xiao Lou, Kaixun Hu, Ang Zhang, Qiyun Sun, Yajing Huang, Huisheng Ai, Mei Guo

**Affiliations:** ^1^ Senior Department of Hematology, the Fifth Medical Center of PLA General Hospital, Beijing, China; ^2^ Chinese PLA Medical School, Chinese PLA General Hospital, Beijing, China; ^3^ Department of Hematology, Strategic Support Force Medical Center, Beijing, China; ^4^ Department of Hematology, Innovvy for Biotechnology Experiments Center, Beijing, China

**Keywords:** myeloma, immunotherapies, immunology, micro-transplant, microchimerism

## Abstract

**Background:**

Despite advances in the treatment of multiple myeloma, a proportion of patients still hardly achieve desired prognosis. Although microtransplant (MST) has proved promising results in treating several hematological malignancies, it has not been studied in multiple myeloma.

**Methods:**

We performed a retrospective analysis of multiple myeloma patients treated with MST at our institution. Their clinical information and outcome measurements were collected. Furthermore, the fluctuation of donor microchimerism after MST, as well as the alteration of immune function before and after MST were analyzed.

**Results:**

Twenty patients receiving MST were enrolled from June 2008, to May 2024, with an overall response rate of 17/20. The 6-year overall survival (OS) and progression-free survival (PFS) rates were 64.7% and 35.3%, respectively, with no graft-versus-host disease or non-relapse mortality. Incidence of controlled fever and Grade I cytokine release syndrome (CRS) was 40.8%. The OS were comparable between groups with age, International Staging System stage, and Mayo Stratification of Myeloma and Risk-Adapted Therapy stage. However, earlier Durie-Salmon stage, disease in VGPR or CR status prior to MST, and an increase in total cycle number of MST were significantly associated with longer OS. Donor microchimerism was detected in all available peripheral blood samples from 14 days to 6 months post-MST. Furthermore, MST resulted in increased proportions of total CD3+ T cells, and CD4+CD8- T cells in peripheral blood, as well as improved CD4:CD8 ratio and increased proportions of Th0 cells.

**Conclusion:**

MST extended PFS and OS, and benefit immune reconstitution in multiple myeloma patients. Therefore, MST is a promising treatment for multiple myeloma, especially those with high-risk cytogenetics.

## 1 Introduction

Multiple myeloma is a hematological malignancy that can be caused by abnormal proliferation of monoclonal plasma cells in the patient’s bone marrow, leading to a series of clinical manifestations (1). Due to the clinical use of new drugs, patients’ survival has significantly improved compared to the past (2). At the same time, autologous hematopoietic stem cell transplantation can effectively prolong progression-free survival and overall survival by improving the bone marrow hematopoietic microenvironment and achieving deep disease remission through myeloablative conditioning. Therefore, single or sequential autologous hematopoietic stem cell transplantation combined with maintenance therapy are currently standard methods for treating multiple myeloma (3).

However, autologous hematopoietic stem cell transplantation is generally only suitable for patients under 65 years (3) or elderly patients with good physical condition. High-dose melphalan can cause adverse reactions such as cardiac toxicity. Additionally, delayed recovery of neutrophils and platelets can increase the risk of severe infection and bleeding (4). Furthermore, factors such as patient’s physical condition and stem cell mobilization effect can affect the quantity and quality of hematopoietic stem cell collection, bringing uncertain factors to subsequent transplantation.

Patients with high-risk genetic factors and short-term relapse after autologous transplantation may benefit from allogeneic hematopoietic stem cell transplantation (5, 6). Although strategies such as reduced-intensity conditioning and post-transplant cyclophosphamide are used to reduce drug toxicity and decrease the incidence of GVHD (7, 8), the higher transplant-related mortality (TRM) still limits the use of allogeneic transplantation for multiple myeloma.

MST is a treatment method combining chemotherapy with the infusion of G-CSF mobilized HLA-mismatched donor peripheral blood stem cells. MST can promote hematopoietic recovery and extend progression-free survival and overall survival for patients with different types of diseases such as acute myeloid leukemia, acute lymphoblastic leukemia, and non-Hodgkin’s lymphoma (9–18). However, its application in MM has not been reported. In this study, we retrospectively analyzed data from 20 patients with MM who received MST treatment, in order to explore new strategies for the treatment of multiple myeloma.

## 2 Methods

### 2.1 Patients and donors

From June 1st, 2008, to May 10th, 2024, patients aged 40-74 years who were diagnosed with multiple myeloma were screened. The diagnosis criteria refer to the International Myeloma Working Group Criteria for Diagnosis of Multiple Myeloma (19), and the prognosis stratification refers to mSMART (20). Patients undergoing MST must meet the following criteria: 1. Voluntary participation and signing of informed consent form; 2. Peripheral blood hematopoietic stem cells provided by healthy donors are available; 3. Diagnosed with multiple myeloma and in initial diagnosis or post-induction phase (PR, VGPR, CR or relapse). Exclusion criteria include: 1. Severe organ dysfunction including NYHA grade III-IV heart failure, liver failure, renal failure, etc.; 2. Severe embolism or thrombotic events; 3. Pregnancy and lactation; 4. Severe infectious diseases such as aspergillosis.

Donor selection criteria include: HLA (human leucocyte antigen) typing ≤7/10 or ≤4/6 matched relative or unrelated donors, physical examination meets the conditions for donating hematopoietic stem cells, informed consent obtained and able to tolerate peripheral blood mononuclear cell collection procedure. Donors with HLA typing containing 3 or more homozygous loci are excluded.

### 2.2 Treatment design

#### 2.2.1 Conditioning regimen for MST

The induction regimens for patients undergoing MST include but are not limited to VCd and VRd. Definitions of response and progression are based on the IMWG criteria (21). The conditioning regimen for MST includes VMd (subcutaneous injection of bortezomib 1.3mg/m^2^ on days 1, 4, 8, and 11, intravenous injection of melphalan 30-40mg/m^2^ on days 1-2, and intravenous injection of dexamethasone 20mg/d on days 1-2, 4-5, 8-9, and 11-12) or VAd (subcutaneous injection of bortezomib 1.3mg/m^2^ on days 1, 4, 8, and 11, intravenous injection of adriamycin 30mg/m^2^ on days 1-2, and intravenous injection of dexamethasone 20mg/d on days 1-2, 4-5, 8-9, and 11-12). Donor G-CSF-mobilized peripheral blood mononuclear cells (GPBMCs) are infused on the 5th day of condition. GVHD, cytomegalovirus infection, and Pneumocystis carinii infection prevention are not performed before or after MST.

#### 2.2.2 Mobilization and apheresis of donor peripheral blood mononuclear cells

After mobilizing with G-CSF for 5 days, the donor’s PBMCs were isolated and collected. Freshly collected donor cells will be used for the first infusion. The other collected cells were divided and frozen in liquid nitrogen. The median numbers of mononuclear, CD34+, and CD3+ cells infused per course were 2.75×10^8^/Kg (range 1.13-3.42×10^8^/Kg), 2.03×10^6^/Kg (range 0.29-4.07×10^6^/Kg), and 1.02×10^8^/Kg (range 0.48-1.60×10^8^/Kg), respectively.

#### 2.2.3 Response criteria and outcome evaluation

OS is defined as the duration from diagnosis to death or last follow-up (until May 2024). PFS is defined as the duration from initial treatment to disease progression or death for any reason. Common Terminology Criteria for Adverse Events (CTCAE) version 5.0 was used to grade cytopenia, infection, fever, CRS, and organ toxicities (22). Neutrophil recovery is defined as the first day of three consecutive days that neutrophils are higher than 0.5×10^9^/L, and platelet recovery is defined as the first day of three consecutive days that platelets are higher than 25×10^9^/L independent of transfusion. The occurrence and grading of acute/chronic GVHD are determined by the IBMTR diagnosis criteria for graft-versus-host disease (23).

#### 2.2.4 Microchimerism assessment

After donor cell infusion, the donor microchimerism was detected using an indel-primer-based real-time PCR method on day +1, day +7, and days after hematopoietic recovery as previously described (18).

#### 2.2.5 Analysis of Immune function and T cell cytotoxicity

Immune function and T lymphocyte cytotoxicity testing were performed using flow cytometry 2-3 weeks before and 2 weeks after the infusion of donor GPBMCs. In the immune function test, helper/inducer T cells, suppressor/cytotoxic T cells, total T lymphocytes, γ/δ T cells, B cells, NK cells, NKT cells, cytotoxic T cells, suppressive T cells, regulatory T cells, Th0 cells, Th1 cells, Th2 cells, Tc0 cells, Tc1 cells, and Tc2 cells were defined as CD3^+^CD4^+^CD8^-^, CD3^+^CD4^-^CD8^+^, CD3^+^CD19^-^, CD3^+^CD4^-^CD8^-^, CD3^-^CD19^+^, CD3^-^CD(16 + 56)^+^, CD3^+^CD(16 + 56)^+^, CD3^+^CD8^+^CD28^+^, CD3^+^CD8^+^CD28^-^, CD4^+^CD25^+^CD127^dim^, CD3^+^CD4^+^IFN-γ^+^IL-4^+^, CD3^+^CD4^+^IFN-γ^+^IL-4^-^, CD3^+^CD4^+^IFN-γ^-^IL-4^+^, CD3^+^CD8^+^IFN-γ^+^IL-4^+^, CD3^+^CD8^+^IFN-γ^+^IL-4^-^, and CD3^+^CD8^+^IFN-γ^-^IL-4^+^, respectively.

#### 2.2.6 Statistical analysis

SPSS 19 software was used for all the statistical analyses. Survival data was analyzed by means of log-rank test and the survival curves were made by the Kaplan-Meier method. The T-test or Wilcoxon-test was used to assess the probability of significant differences in survivals. Statistical significance was defined as P <0.05.

#### 2.2.7 Ethics approval

This study protocol has been reviewed by our center’s Human Ethics Committee and is in accordance with the Declaration of Helsinki. All patients and donors signed an informed consent form before MST.

## 3 Results

### 3.1 Patient characteristics

This study included a total of 20 patients. **Table 1** summarizes the characteristics and risk stratification of the enrolled patients. Of the 20 patients, 9 had high-risk genetic features. 14 were in VGPR or CR status, 2 in PR, and 3 in relapse status before MST. One patient was newly diagnosed who had not received any chemotherapy. 17 patients received GPBMC infusion from related donors, and 3 patients from unrelated donors. The median number of MST cycles received by each patient was 3, with a maximum of 13. The median single cell infusion doses of MNC, CD34, and CD3 were 2.75×10^8^/Kg, 2.03×10^6^/Kg, and 1.02×10^8^/Kg, respectively.

**Table 1:** Characteristics of patients.

Parameter	MST (n=20)
Age,median years (range)	57 (40-74)
Male sex,n (%)	13 (65)
DS stage I-II,n (%)	13 (65)
DS stage III,n (%)	7 (35)
ISS I-II,n (%)	19 (95)
ISS III,n (%)	1 (5)
Donor type,n (%)	
Related	17 (85)
Unrelated	3 (15)
MM type,n (%)	
IgG	15 (75)
IgA	2 (10)
IgD	2 (10)
Light chain	1 (5)
Molecular cytogenetic abnormalities, positive,n (%)	
del13q	3 (15)
del17p	3 (15)
t (4;14)	2 (10)
1q gains	4 (20)
SMART,n (%)	
standard risk	11 (55)
high risk	9 (45)
Response prior to MST,n (%)	
VGPR and CR	14 (70)
PR	2 (10)
Relapse	3 (15)
untreated	1 (5)
Median cycle of MST, (Range)	3 (1-13)
Dose of DCI	
Median MNC (range)/×10^8^/Kg	2.75 (1.13-3.42)
Median CD34 (range)/×10^6^/Kg	2.03 (0.29-4.07)
Median CD3 (range)×10^8^/Kg	1.02 (0.48-1.6)

MST, microtransplant; DS, Durie-Salmon; ISS, International staging system; MM, multiple myeloma; Ig, immunoglobulin; del, deletion of chromosome; 1q Gains, chromosome 1q21 gains; t, translocation; SMART, Mayo Stratification of Myeloma and Risk-Adapted Therapy.

### 3.2 Hematopoietic recovery and toxicity

**Figure 1:**
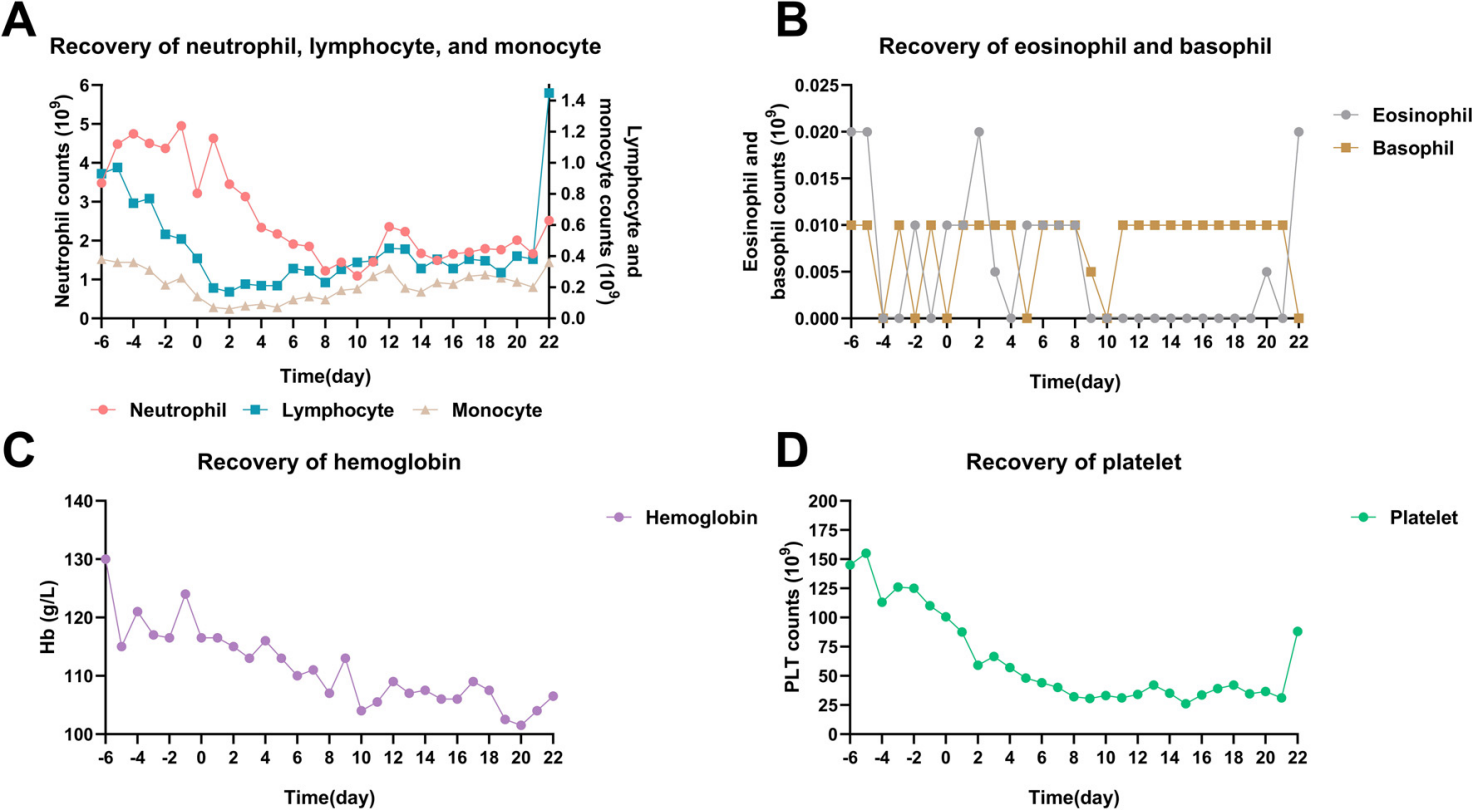
Time courses of hematopoietic recovery in total 76 cycles of MST. **(A)** Neutrophil, lymphocyte, and monocyte recovery. **(B)** Eosinophil and basophil recovery. **(C)** Hemoglobin recovery. **(D)** Platelet recovery. MM patients received donor cell infusion at day 0, and the median number was used to represent the blood cell counts.

A total of 76 cycles of MST were performed on 20 patients. The hematopoietic recovery and adverse events (AEs) are shown in **Table 2**. Grade IV lymphocytopenia was relatively common (59.2%), while Grade III-IV anemia was not pronounced (8%). The overall incidence of Grade IV neutropenia and Grade IV thrombocytopenia was 27.6% and 38.1%, respectively. Six patients did not experience Grade IV neutropenia or thrombocytopenia during treatment of MST. In the remaining 14 patients, the median duration of Grade IV neutropenia and thrombocytopenia was 3.5 days and 9 days, respectively. Total time courses of hematopoietic recovery, including neutrophils, lymphocytes, monocytes, eosinophils, basophils, hemoglobin, and platelet, are presented in **Figure 1**. The degree of bone marrow suppression was relatively mild (24). No immature neutrophils or other peripheral blood cells were observed in 76 cycles of MST.

**Table 2:** Hematopenia and adverse events.

Event	Grade I	Grade II	Grade III	Grade IV
Neutrophil count decreased	14 (18.4)	10 (13.2)	21 (27.6)	21 (27.6)
Lymphocyte count decreased	2 (2.6)	8 (10.5)	21 (27.6)	45 (59.2)
Anemia	29 (38.2)	23 (30.3)	6 (8%)	0
Platelet count decreased	13 (17.1)	12 (15.8)	18 (23.7)	29 (38.1)
Fever	23 (30.2)	8 (10.5)	0	0
Rash	2 (2.6)	0	0	0
Gastrointestinal disorder	3 (3.9)	1 (1.3)	0	0
Liver function injury	2 (2.6)	0	0	0
Kidney dysfunction	0	0	0	0
Infection	0	0	4 (5.3)	0
CRS	31 (40.8)	0	0	0
GVHD	0	0	0	0
Median time of ANC<0.5×10^9^/L, (range)	3.5d (1-14)			
Median time of PLT<25×10^9^/L, (range)	9d (1-21)			

ANC, absolute neutrophil count; CRS, cytokine release syndrome; GVHD, graft-versus-host disease.

**Figure 2:**
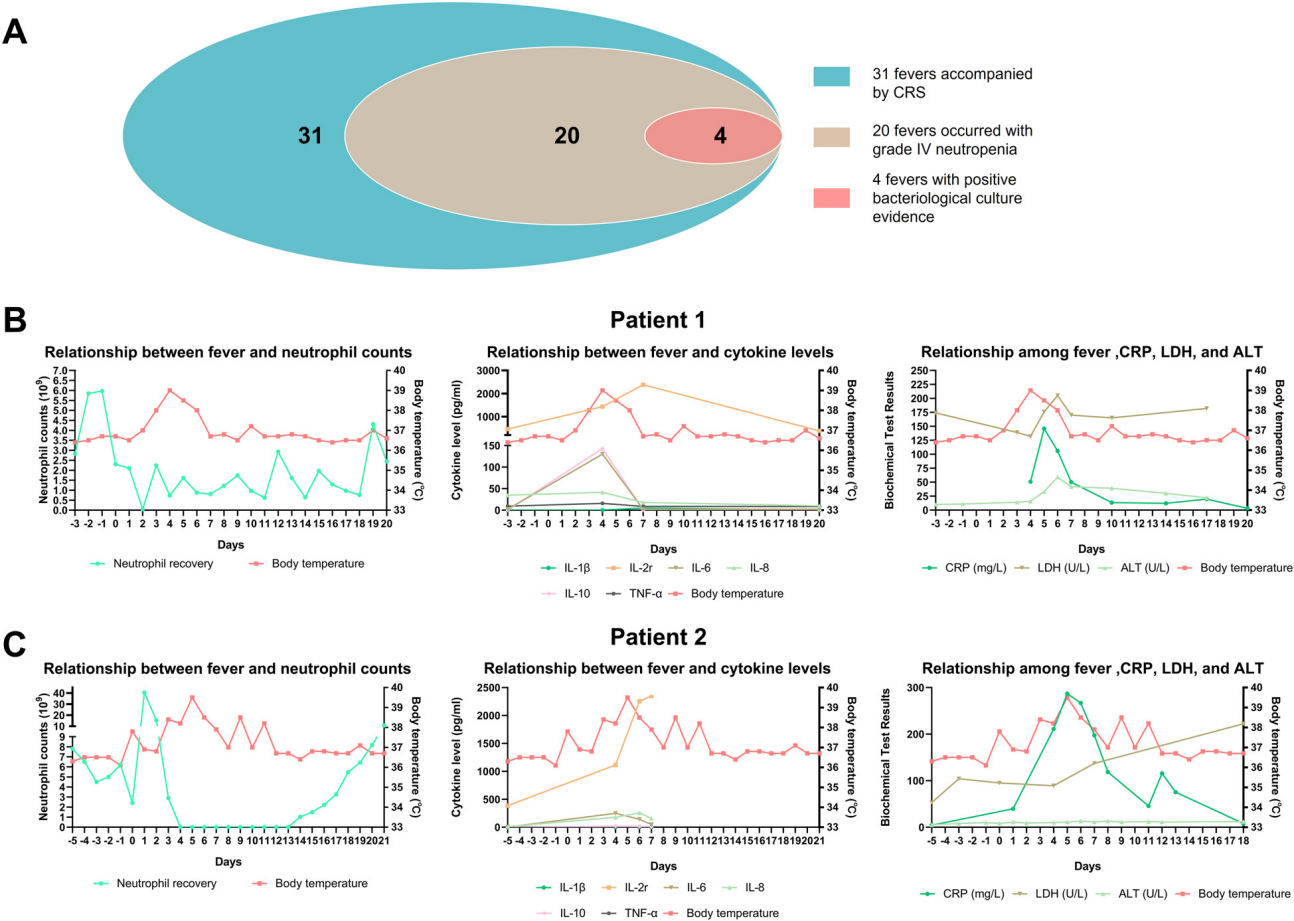
The discrimination of fever causes. **(A)** Overview of fever condition; **(B, C)** The relationship between fever and neutrophil counts, inflammatory cytokine levels, as well as other biochemical test results in two patients.

Fever and donor cell infusion-related cytokine release syndrome (CRS) were the most common AEs. Grade I and Grade II fevers were observed in 23 (30.2%) and 8 (10.5%) cases, respectively. All fevers could not be ruled out as being related to CRS. Among them, 20 fever events occurred during the period of Grade IV neutropenia, and 4 of these had definitive positive blood culture results, indicating that fever was also associated with severe infection (**Figure 2A**). Here, two typical cases were shown to distinguish the causes of fever. Patient 1 and Patient 2 both experienced febrile neutropenia three to five days after donor cell infusion, and the increase of C-reactive protein (CRP), lactate dehydrogenase (LDH), alanine aminotransferase (ALT), and cytokines such as interleukin-2r (IL-2r), IL-6, IL-8, were observed (**Figures 2B, C**). However, these indicators could elevate both in CRS and infection. Patient 2 had a positive blood culture result that indicated an infection with *Staphylococcus hominis*, accompanied by significantly enhanced CRP. Despite the use of carbapenem antibiotics and glucocorticoid, fever was hardly controlled until the neutrophil recovered. Compared to Patient 2, fever in Patient 1 was effectively controlled after the administration of third-generation cephalosporins and glucocorticoid, and there was insufficient evidence of infection based on clinical manifestations, pathogenic microorganism testing, as well as imaging examinations. Therefore, fever of Patient 1 was more likely associated with CRS, while Patient 2’s fever had a close relationship with both CRS and infection.

Despite the occurrence of CRS after donor cell infusion, accompanied by mild clinical manifestations such as skin rash (2.6%), liver function injury (2.6%), and gastrointestinal disorder (3.9%), the absence of a high proportion of donor chimerism after MST suggested that these immune responses induced by donor cell infusion were probably not sufficient to cause GVHD.

In the long-term follow-up observations, hypogammaglobulinemia had the highest incidence (45%) among the total infection events and immune-related adverse events, followed by lower respiratory infection (35%), intestinal infection (30%), upper respiratory infection (30%), mucosal infection (25%), and shingles (20%) (**Table 3**).

**Table 3:** Total infection and immune AE in long-term follow-up.

Infection and immune AE	MST (n=20)
Hypogammaglobulinemia, n (%)	9 (45)
Lower respiratory infection, n (%)	7 (35)
Intestinal infection, n (%)	6 (30)
Upper respiratory infection, n (%)	6 (30)
Mucosal infection, n (%)	5 (25)
Shingles, n (%)	4 (20)
EB viremia, n (%)	2 (10)
Wheezing, n (%)	1 (5)
Urinary tract infection, n (%)	1 (5)

AE, adverse events; EB, Epstein-Barr.

### 3.3 ORR, OS, and PFS

Among Fourteen patients in VGPR/CR status, twelve maintained VGPR/CR after MST. One of two patients in PR status achieved VGPR/CR after MST. All three patients in PD status achieved PR/VGPR/CR. One previously untreated patient achieved CR after one cycle of MST, resulting in an ORR of 17/20 (85%) for all 20 patients.

**Figure 3:**
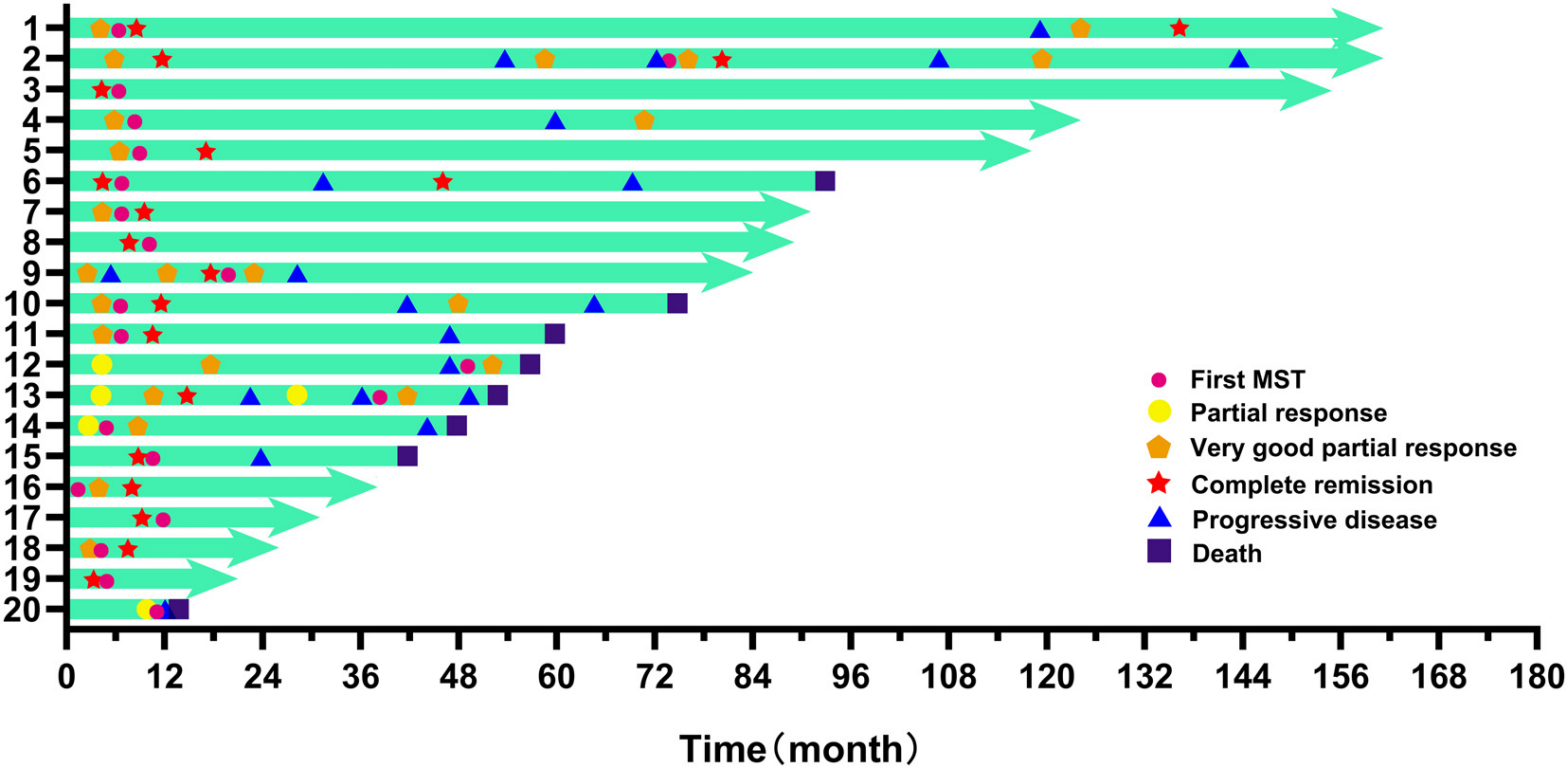
Disease response in the 20 MST-treated patients.

**Figure 4:**
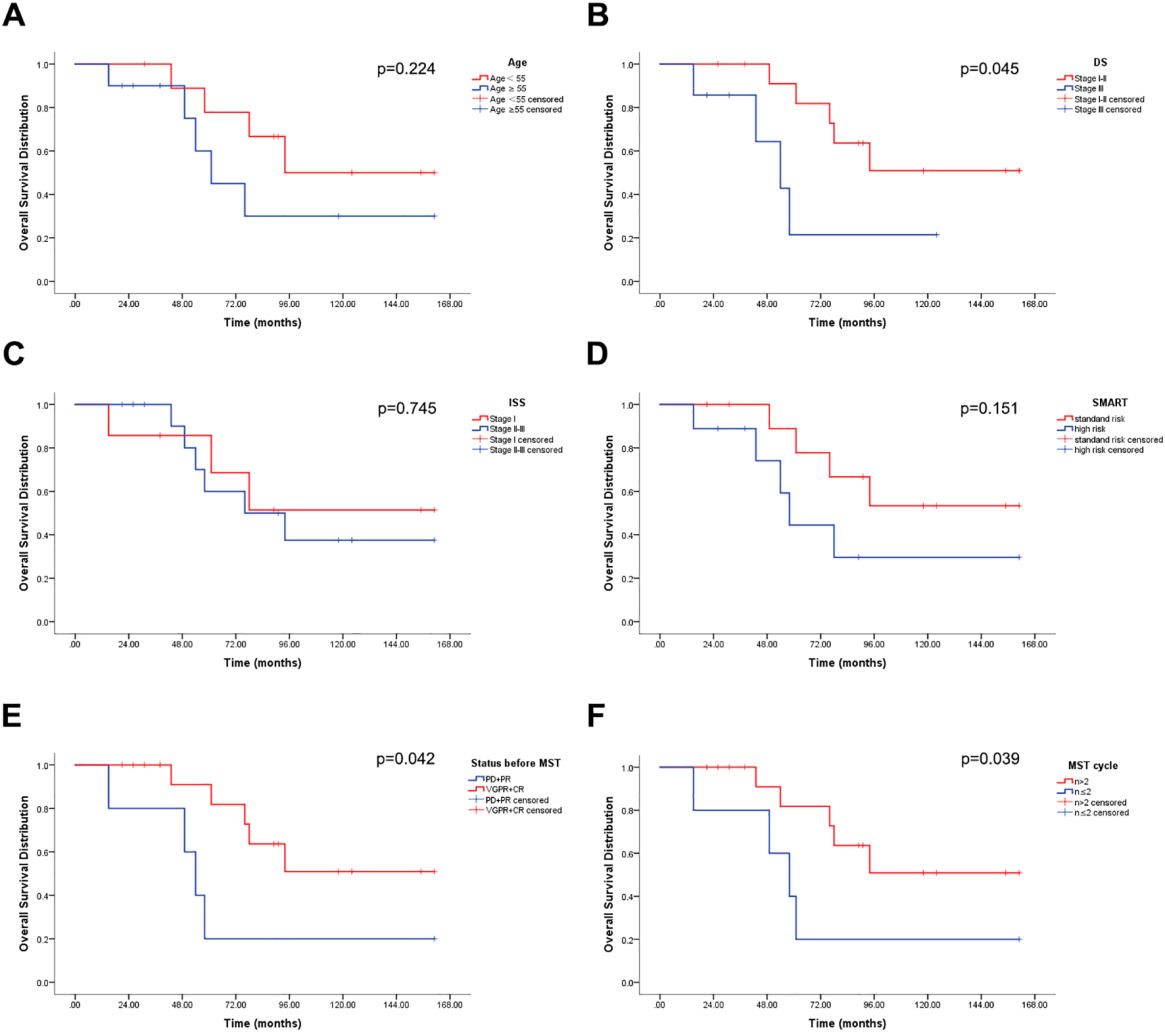
Overall survival (OS) distribution (n = 20). **(A)** OS stratified by age. **(B)** OS stratified by DS stage. **(C)** OS stratified by ISS stage. **(D)** OS stratified by SMART. **(E)** OS stratified by disease status before MST. **(F)** OS stratified by number of MST cycles.

The overview of 20 patients including the time of first cycle of MST, efficacy, and survival were shown in **Figure 3**. The median OS was 91 months (15-161), with a 1-year and 3-year OS of 100% and 94.1%, respectively (**Table 4**), and a 6-year OS of 64.7%. Subgroup analysis based on age, DS stage, ISS stage, SMART stage, pre-MST disease status, and number of sequential MST showed that age, ISS stage, and SMART stage had no significant correlation with 6-year OS (p=0.224, 0.745, 0.151) (**Figures 4A, C, D**), while DS stage showed a significant correlation with prognosis (P=0.045) (**Figure 4B**). Patients in VGPR and CR before MST had a better prognosis than those in PR and PD states (p=0.042) (**Figure 4E**). More cycles of MST (n>2) were associated with longer OS compared to two or fewer (n ≤ 2) (P=0.039) (**Figure 4F**).

**Table 4:** Outcomes of Patients.

Parameter	MST(n=20)
NRM,n(%)	0
Median OS(range)	91(15-161)
Median PFS(range)	39(2-147)
3-year OS, No. of Events(%)	16(94.1)
3-year PFS, No. of Events(%)	11(64.7)
6-year OS, No. of Events(%)	11(64.7)
6-year PFS, No. of Events(%)	6(35.3)
Age (year)	
≥55	7(77.8)
≥55	4(50)
DS stage	
I	1(50)
II	9(90)
III	1(20)
ISS stage	
I	5(71.4)
II	6(60)
SMART	
standard risk	7(77.8)
high risk	4(50)
Status before MST	
PD and PR	1(20)
VGPR and CR	10(83.3)
MST cycle	
>2	10(83.3)
≤2	1(20)
Age (year)	
<55	4(44.4)
≥55	2(25)
DS stage	
I	1(50)
II	5(50)
III	0
ISS stage	
I	4(57.1)
II	2(20)
SMART	
standard risk	4(44.4)
high risk	2(25)
Status before MST	
PD and PR	0
VGPR and CR	6(50)
MST cycle	
>2	5(41.7)
≤2	1(20)

NRM, non-relapse mortality; OS, overall survival; PFS, progression-free survival.

**Figure 5:**
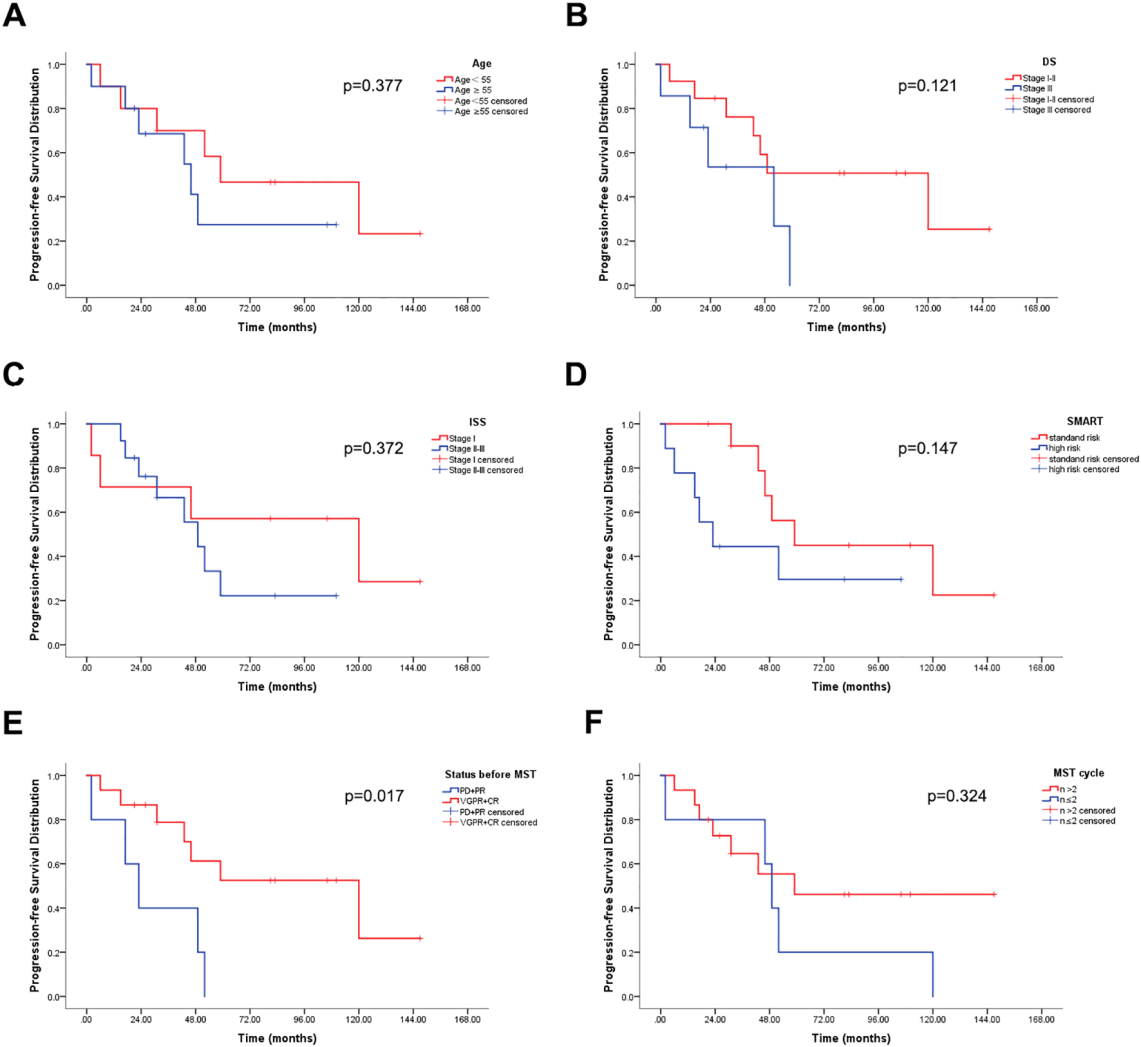
Progression-free survival (PFS) distribution (n = 20). **(A)** PFS stratified by age. **(B)** PFS stratified by DS stage. **(C)** PFS stratified by ISS stage. **(D)** PFS stratified by SMART. **(E)** PFS stratified by disease status before MST. **(F)** PFS stratified by number of MST cycles.

The median PFS for patients receiving MST was 39 months (2-150), with an overall 6-year PFS of 35.3%. Age, DS staging, ISS staging, SMART staging, and the number of MST cycles were not significantly correlated with PFS (p=0.377, 0.121, 0.372, 0.147, 0.324), whereas disease status before MST was significantly correlated with PFS (p=0.017) (**Figure 5**).

Among the sixteen IgG myeloma patients, the median PFS was 46 months (2-150) and the median OS was 89 months (15-161). For the two IgD patients, PFS was 20 months and 110 months, and OS was 43 months and 118 months, respectively. One IgA patient in disease relapse status achieved CR with an OS of 161 months after MST. Another IgA patient had a total disease course of 26 months, a PFS of 24 months, and is currently still in CR.

### 3.4 NRM

No NRM occurred.

### 3.5 Univariate and multivariate analyses

We performed univariate and multivariate regression analyses to identify potential prognostic factors on OS and PFS, including patient age, gender, DS stage, ISS stage, SMART stage, disease status before MST, number of MST cycles, and infused cell dose in MST (MNC, CD34, CD3). Multivariate regression analysis revealed that more MST cycles (≥2) were significantly associated with longer OS (p=0.043) (**Table 5**).

**Table 5:** Univariate and multivariate analyses of factors prognostic for OS and PFS (n=20).

Variables	6year OS				6year PFS			
	Univariate analysis		Multivariate analysis		Univariate analysis		Multivariate analysis	
	%	P	HR (95%CI)	P	%	P	HR (95%CI)	P
Age								
<55	80	0.342	4.411 (0.48-40.531)	0.19	50	0.661	-	-
≥55	60		1	-	40		-	-
Gender								
Male	84.6	0.058	2.638 (0.361-19.263)	0.339	46.2	0.89	-	-
Female	42.9		1	-	42.9		-	-
DS								
I-II	84.6	0.058	7.703 (0.876-67.736)	0.066	53.8	0.291	1.471 (0.364-5.947)	0.588
III	42.9		1	-	28.6		1	-
ISS								
I	71.4	0.921	-	-	57.1	0.435	-	-
II-III	69.2		-	-	38.5		-	-
SMART								
standard risk	81.8	0.214	7.391 (0.539-101.305)	0.134	54.5	0.355	1.807 (0.469-6.955)	0.39
high risk	55.6		1	-	33.3		1	-
Status before MST								
≤PR	20	0.006	3.824 (0.19-76.902)	0.381	0	0.023	0.293 (0.044-1.971)	0.207
≥VGPR	86.7		1	-	60		1	-
MST cycle								
>2	86.7	0.006	6.499 (1.058-39.915)	0.043	53.3	0.206	0.661 (0.125-3.501)	0.626
≤2	20		1	-	20		1	-
MNC infused in the first cycle								
≥3×10^8^/Kg	66.7	0.836	-	-	33.3	0.503	-	-
<3×10^8^/Kg	71.4		-	-	50		-	-
CD34 infused in the first cycle								
≥2×10^6^/Kg	70	1	-	-	40	0.661	-	-
<2×10^6^/Kg	70		-	-	50		-	-
CD3 infused in the first cycle								
≥0.8×10^8^/Kg	70	1	-	-	50	0.661	-	-
<0.8×10^8^/Kg	70		-	-	40		-	-

HR, hazard ratio; MNC, mononuclear cell.

### 3.6 Microchimerism

**Figure 6:**
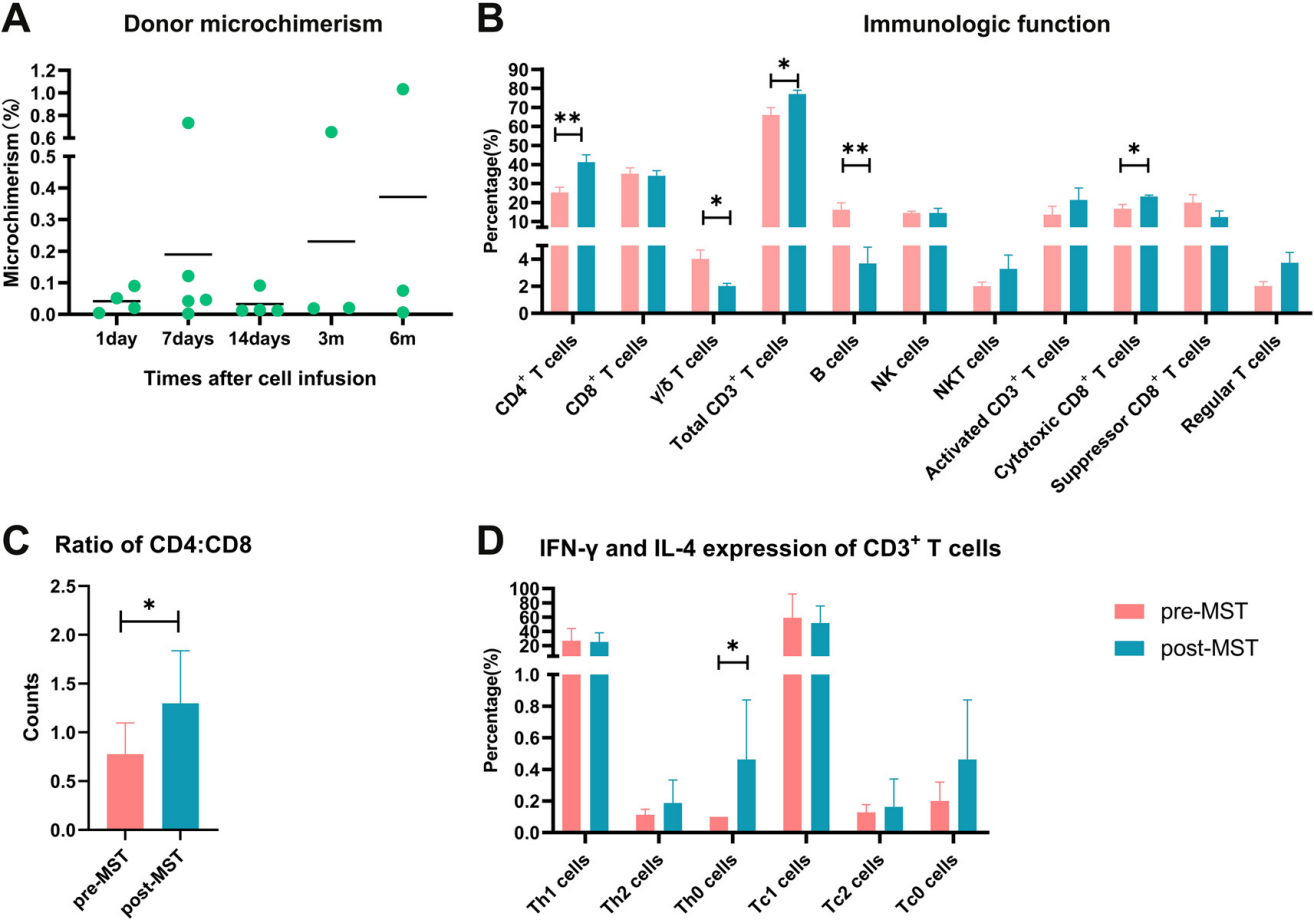
Overview of donor microchimerism, and the evaluation of immune function in MST-treated patients (n=20). **(A)** Donor peripheral blood microchimerism after each infusion determined by an Indel-primer-based real-time PCR method. **(B)** Alternation of immune function before and after MST. **(C)** Ratio of CD3^+^CD4^+^ and CD3^+^CD8^+^ T cells. **(D)** Expression of IFN-γ and IL-4 in CD3^+^ T cells. The symbol “*” means the P<0.05, “**” means P<0.01.

Among these 20 patients, 3 patients had totally 19 available peripheral blood samples for donor microchimerism tests, and all 3 patients had detectable donor microchimerism (<1.2%) after MST. The chimerism levels fluctuated between 0.012-0.091% on day 1, 0.002-0.735% on day 7, 0.012-0.091% on day 14, 0.02-0.65% at 3 months, and 0.006-1.034% at 6 months, respectively after cell infusion (**Figure 6A**). The disease course of these 3 patients was 21, 26, and 31 months, respectively, and they are currently in sustained remission at last follow-up.

### 3.7 Immune reconstitution

We tested lymphocyte subsets and tumor cell-specific T cell killing function on 5 patients before and after MST. The results showed that the proportions of CD4+ T lymphocytes, total CD3+ T lymphocytes, and cytotoxic CD8+ T cells in peripheral blood after MST were 41.3 ± 10.0%, 77.0 ± 5.8%, and 23.2 ± 2.1%, respectively, which were significantly higher than those before MST of 25.4 ± 7.2%, 66.0 ± 10.9%, and 16.8 ± 6.2% (p=0.005, 0.025, 0.015) (**Figure 6B**). The proportions of NKT and NK cells showed tendency to increase after MST, but with no statistical difference (p=0.993, 0.241). The proportions of γ/δ T and B cells decreased after MST (p=0.012, 0.005). The CD4/CD8 ratio after MST was 1.30 ± 0.54, which was significantly higher than that before MST of 0.78 ± 0.32 (p=0.048) (**Figure 6C**). Th0 cells was 0.19 ± 0.15% after MST, which was higher than that before MST of 0.11 ± 0.04% (p=0.017) (**Figure 6D**).

## 4 Discussion

Although many effective treatment methods are available, multiple myeloma remains an incurable disease. No matter how much the progression-free survival and overall survival are extended, it seems difficult to avoid disease relapse. In order to achieve deep disease remission and prolong survival, classical regimens combining with immunotherapies such as MST are feasible measures. In addition, the relapse of multiple myeloma is closely related to immune imbalance, therefore, modulating immune function through immunotherapy is also particularly important.

The anti-tumor effect of DLI in the treatment of multiple myeloma has been validated (25, 26). In this study, the effectiveness of MST for the treatment of multiple myeloma was demonstrated for the first time. The ORR of patients receiving MST reached 85%, and the median PFS and OS were 39 months and 91 months, respectively. Earlier DS staging, disease in VGPR or CR status before MST, and an increase in total cycle number of MST suggest better prognosis. The 3-year OS of high-risk patients with adverse chromosomal changes after autologous transplantation was 48%-77% (27). In our study, the 3-year and 6-year OS rates of high-risk patients in SMART staging were 87.5% and 37.5%, respectively, and the median OS was 61 months. Meanwhile, the stratification of SMART risk level was not significantly correlated with the prognosis of MST, suggesting that MST may improve the prognosis of patients with high-risk cytogenetic abnormalities through immunotherapy.

In autologous hematopoietic stem cell transplantation, intensive chemotherapy such as high-dose melphalan is recommended for achieving deep disease remission, which may lead to prolonged bone marrow suppression and life-threatening complications such as severe infections. The TRM of autologous hematopoietic stem cell transplantation is 0-10% (28–31). In contrast, MST uses a lower dose of melphalan, with relatively mild suppression of hematopoiesis and a lower risk of complications. In this clinical study with small sample size, no NRM occurred. Additionally, the incidence and severity of cytokine release syndrome (CRS) associated with lymphocyte infusion is also important to assess safety. In the clinic trials of B cell maturation antigen (BCMA) targeting CAR-T cells, the initial symptom of CRS is fever, followed by low hypotension, hypoxia, and end organ toxicity (32, 33). In this study, the transient increase of inflammatory cytokines and CRP coupled with fever was also observed. Although the total occurrence rate of CRS was comparable with that in CAR-T cell therapy, it is relatively mild (only in Grade I) and controllable. No acute or chronic GVHD was detected, which may result from the absence of high-proportion donor engraftment. Therefore, MST is safer compared to autologous hematopoietic stem cell transplantation and CAR-T cell therapy.

Meanwhile, the incidence of Grade IV neutropenia and thrombocytopenia in MST was 27.6% and 38.1%, respectively, which was lower than the reported results of melphalan-containing chemotherapy (34–36). Among fourteen patients, the median duration of Grade IV neutropenia and thrombocytopenia was 3.5 days and 9 days, respectively. The other six patients did not experience severe neutropenia or thrombocytopenia during treatment of MST. Milder bone marrow suppression and faster hematopoietic recovery may be related to the infusion of donor G-CSF mobilized MNCs (12, 17).

Interestingly, donor microchimerism was detected in the peripheral blood even after six months following donor MNC infusion, although the level of microchimerism varied greatly among different patients. Previous studies also demonstrated that the longest duration of donor microchimerism was 1020 days (13). Although microchimerism was detectable in all three patients with available samples, the lack of peripheral samples for testing in the remaining 17 patients leaves insufficient evidence to confirm the presence or absence of donor microchimerism in those cases. Given this small sample size, a direct comparative analysis of OS, PFS, AEs, and immune reconstitution between the three microchimerism-positive patients and the rest was not performed. The association between the proportion of microchimerism and the prognosis still needs further investigation.

During the occurrence and progression of MM, patients experience dysfunction and exhaustion of T cells and suppression of the bone marrow microenvironment (37). In this clinical study, recipients were in an abnormal immune functional state with a reversed CD4/CD8 ratio before treatment, and the proportion of Th0 cells, that belong to a kind of naive T cells, was low. This suggests that the occurrence of multiple myeloma may be related to mechanisms such as immune dysfunction and immune escape. After MST, the proportion of CD4+ T lymphocytes and total CD3+ T lymphocytes in peripheral blood increased significantly, the CD4/CD8 ratio also increased, as well as the proportion of Th0 cells, indicating that the immune balance of patients was partially recovered, T cell cytotoxic capability was improved, immune reconstitution was accelerated, and abnormal immune homeostasis was partially corrected. This partly explains that the prognosis of patients receiving MST is not inferior to those receiving ASCT, and even better in overall survival (27). We also observed a significant decrease in the proportion of γ/δ T cells after MST. Previous reports have shown that γ/δ T cells can exert anti-tumor effects (38) or promote tumor cell proliferation in the process of tumor development (39, 40). Therefore, the role of γ/δ T cells in MST still needs to be further studied.

In summary, microtransplant extended PFS and OS and improved immune function, which provided an alternative treatment option for patients with multiple myeloma, especially those with high-risk cytogenetics. However, prospective studies with larger sample sizes are still needed.

## Data Availability

The original contributions presented in the study are included in the article/supplementary material. Further inquiries can be directed to the corresponding authors.
